# Supplementary material for: BAFF-neutralizing interaction of belimumab related to its therapeutic efficacy for treating systemic lupus erythematosus
Source: Nat Commun. 2018 Mar 23;9:1200. doi: 10.1038/s41467-018-03620-2 (PMC5865148; doi:10.1038/s41467-018-03620-2)
Supplement: Supplementary file 1 — Supplementary Information(PDF 413 kb) [file 41467_2018_3620_MOESM1_ESM.pdf]

SUPPLEMENTARY INFORMATION

BAFF-neutralizing interaction of belimumab related to its therapeutic efficacy for treating systemic lupus erythematosus

Shin et al.

SUPPLEMENTARY FIGURES

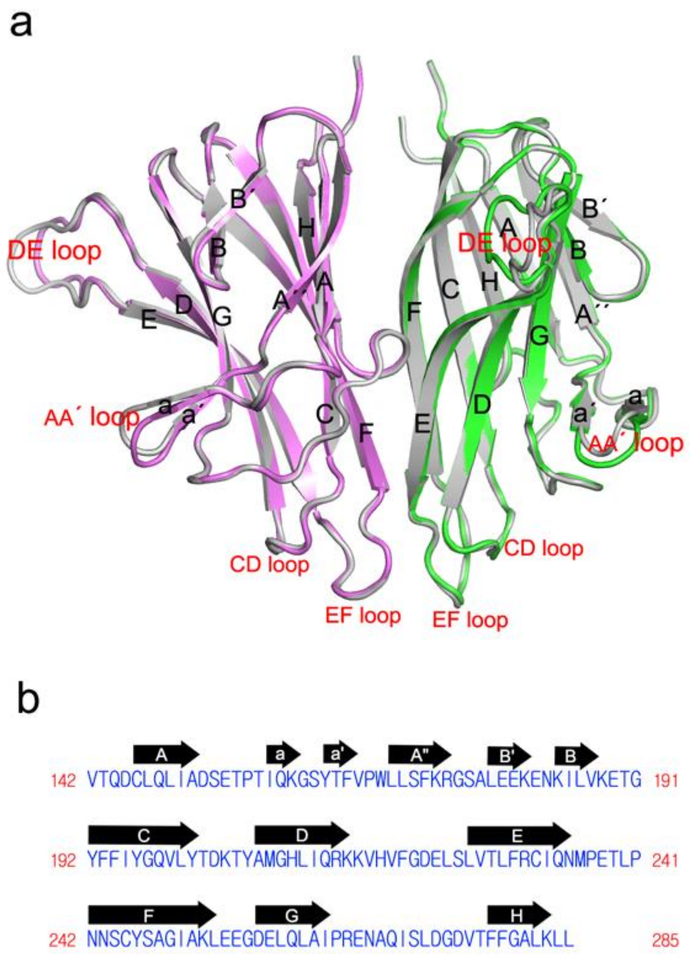

Supplementary Figure 1. Canonical designation of the strands and loops within BAFF.

(**a**) Structural comparison of the BAFF trimers extracted from the BAFF-belimumab complex (purple and green) and the BAFF 60-mer (grey). Only two neighboring protomers in a BAFF trimer are shown for clarity. (**b**) Amino acid sequence of soluble BAFF. The strands in BAFF are denoted with arrows above the sequence.

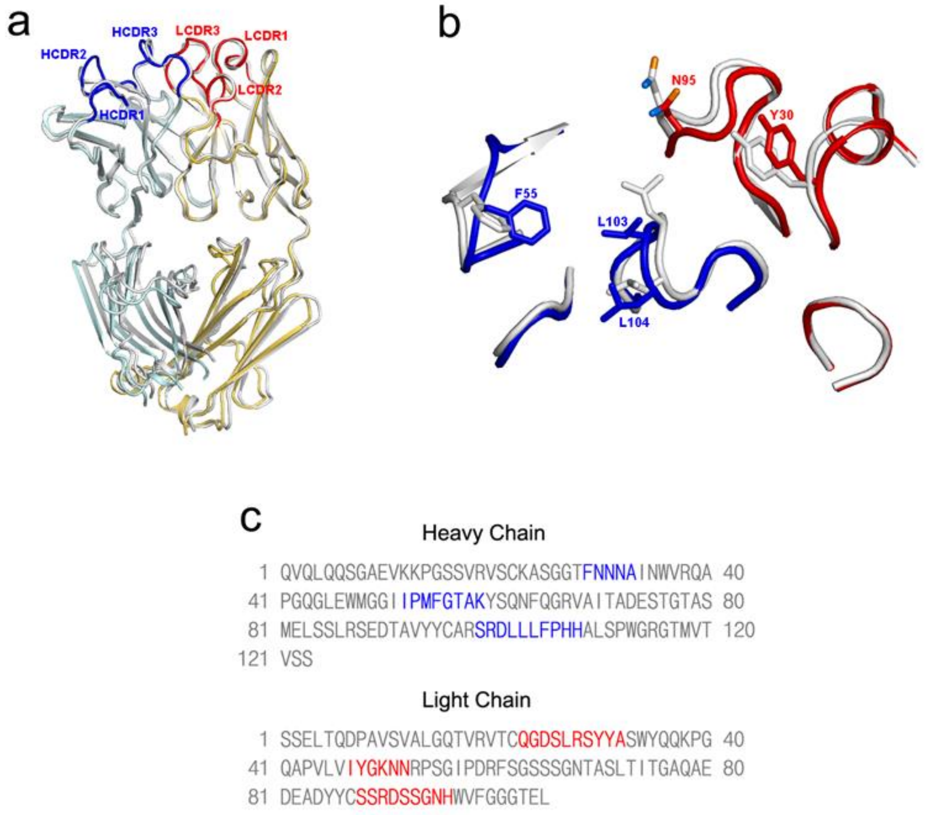

Supplementary Figure 2. Structural comparison of belimumab Fab before and after binding to BAFF.

(**a**) Superposition of the Fv region of free belimumab Fab (grey) onto that of belimumab in complex with BAFF (heavy chain: cyan; light chain: yellow). Belimumab CDRs in the BAFF-belimumab complex are colored blue and red. (**b**) Close-up view of the structural differences of the CDRs in **a**. (**c**) Amino acid sequence of the belimumab Fv region. The residues of HCDRs and LCDRs are colored blue and red, respectively.

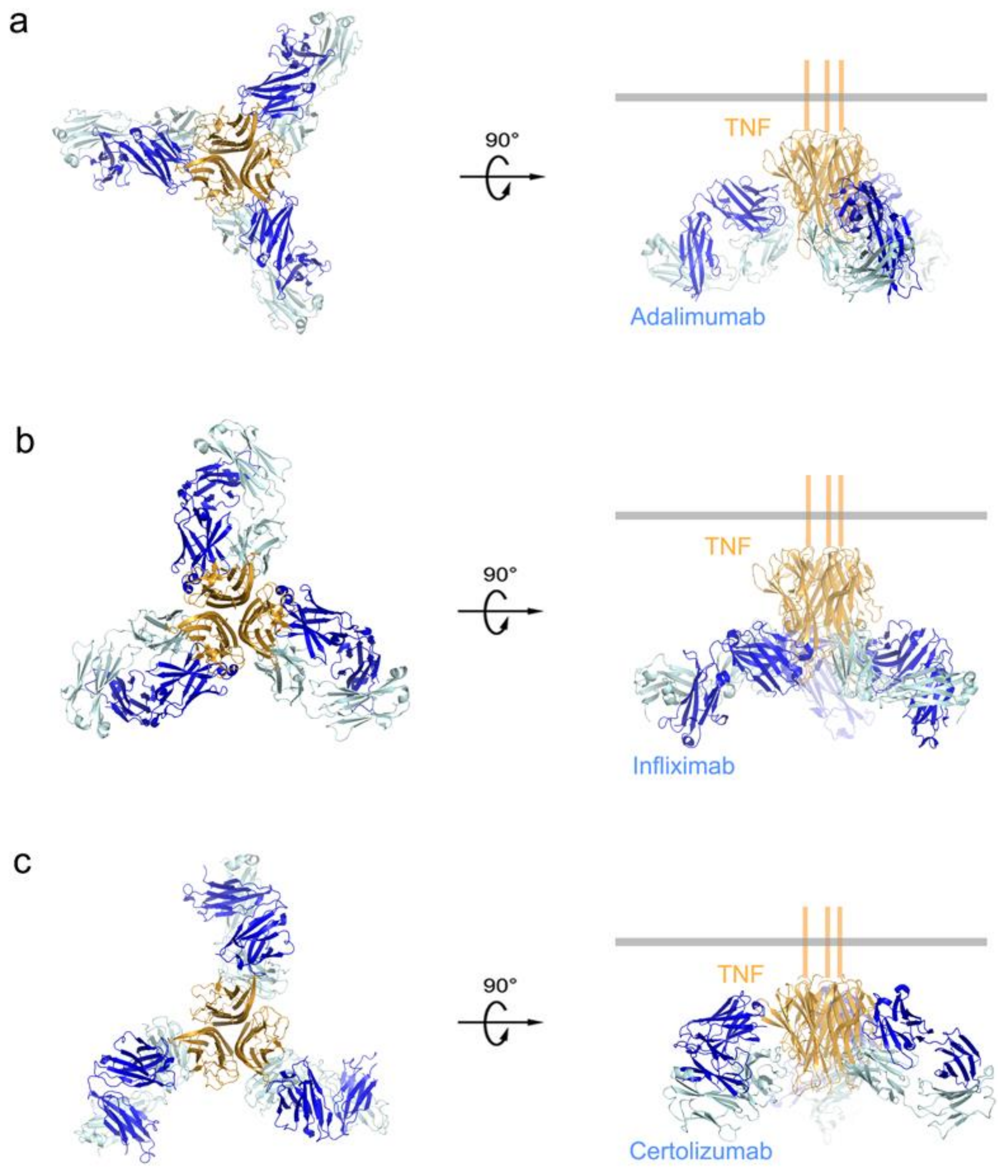

Supplementary Figure 3. Structures of TNF in complex with anti-TNF antibodies.

(**a**) The structure of the TNF-adalimumab Fab complex (PDB ID code 3WD5). (**b**) The structure of the TNF-infliximab Fab complex (PDB ID code 4G3Y). (**c**) The structure of the TNF-certolizumab Fab complex (PDB ID code 5WUX). The TNF trimers are colored yellow, and the heavy chains and light chains of the antibodies are colored blue and cyan, respectively. The grey bars indicate putative membranes of TNF-producing cells if the TNF trimers are in their membrane-bound form.

Supplementary Table 1. Binding kinetics of BAFF WT and mutants with belimumab Fab.

BAFF	k_{on} ($M^{-1} \cdot s^{-1}$)	k_{off} (s^{-1})	K_D (M)
**WT**	$1.001 \pm 0.034 \times 10^{5}$	$9.962 \pm 0.339 \times 10^{-5}$	$9.955 \pm 0.332 \times 10^{-10}$
**Y163A**	$9.442 \pm 0.412 \times 10^{4}$	$1.367 \pm 0.060 \times 10^{-4}$	$1.448 \pm 0.065 \times 10^{-9}$
**Y206A**	$5.561 \pm 0.644 \times 10^{5}$	$7.831 \pm 0.892 \times 10^{-3}$	$1.408 \pm 0.159 \times 10^{-8}$
**D222A**	$8.220 \pm 0.445 \times 10^{4}$	$3.570 \pm 0.182 \times 10^{-4}$	$4.343 \pm 0.237 \times 10^{-9}$
**L224A**	$1.533 \pm 0.160 \times 10^{5}$	$1.191 \pm 0.122 \times 10^{-3}$	$7.770 \pm 0.788 \times 10^{-9}$
**R231A**	$4.501 \pm 0.225 \times 10^{4}$	$1.283 \pm 0.064 \times 10^{-4}$	$2.850 \pm 0.143 \times 10^{-9}$
**I233A**	$1.243 \pm 0.078 \times 10^{5}$	$3.278 \pm 0.189 \times 10^{-4}$	$2.638 \pm 0.152 \times 10^{-9}$
**L240A**	$1.006 \pm 0.043 \times 10^{5}$	$1.453 \pm 0.061 \times 10^{-4}$	$1.445 \pm 0.060 \times 10^{-9}$
**N242A**	$1.367 \pm 0.054 \times 10^{5}$	$1.673 \pm 0.066 \times 10^{-4}$	$1.223 \pm 0.048 \times 10^{-9}$
**R265A**	$1.476 \pm 0.070 \times 10^{4}$	$5.590 \pm 0.242 \times 10^{-5}$	$3.787 \pm 0.169 \times 10^{-9}$
**E266A**	$1.047 \pm 0.061 \times 10^{5}$	$2.452 \pm 0.142 \times 10^{-4}$	$2.342 \pm 0.139 \times 10^{-9}$

Supplementary Table 2. Gene cloning information.

**BAFF (aa 134-285)**
**Cloning vector:** pET-28a (*Nde*I, *Xho*I)<br>**Nucleotide sequence:**<br>gccgttcagggtccagaagaaacagtcactcaagactgcttgcaactgattgcagacagtgaaacaccaactatacaaaaaggatc<br>ttacacatttgttccatggcttctcagctttaaaaggggaagtgccctagaagaaaaagagaataaaatattggtcaaagaaactggtt<br>actttttatatatggtcaggttttatatactgataagacctacgccatgggacatctaattcagaggaagaaggtccatgtctttgggga<br>tgaattgagtctggtgactttgtttcgatgtattcaaaatatgcctgaaacactacccaataattcctgctattcagctggcattgcaaaa<br>ctggaagaaggagatgaactccaacttgcaataccaagagaaaatgcacaaatatcactggatggagatgtcacatttttggtgcat<br>tgaaactgctgtga

**Belimumab Fab**
**Cloning vector:** pBAD-TOPO (TA cloning)<br>**Nucleotide sequence:**<br>atgaaaaagaatatcgcatttcttcttgctagcatgttcgtttttctattgctacaaacgcatacgcttcatctgaacttactcaagatcca<br>gctgtaagcgtggccttgggtcaaacagttcgcgttacatgtcaaggtgattccctgcgtagttattatgcaagttggtaccaacagaa<br>acccggtcaggcaccggtcctggtgatttatggtaaaaataaccgtccgtcagggattcctgatcgtttttcaggtagtagctccggta<br>atacggcctcgttaaccatcaccggtgctcaggcagaagatgaagcggattattattgctctagcagagattcttcaggaaatcattg<br>ggtttttggtggaggtactgagctgactgtcctgggtcaaccaaaggctgccccaagtgttacattgttccctccgagctctgaagag<br>ttacaagcgaataaagccacgctggtgtgcttaatttcagatttttatcctggagcggtgactgtcgcatggaaggctgattcttctccg<br>gttaaagccggcgtagaaactaccacaccgagcaaacagagcaacaataaatatgcagctagtagttatctgtcacttacacccga<br>acagtggaaaagtcatcgttcgtactcctgtcaggttacccacgaaggttccacggtagagaaaactgttgcacctaccgaatgttca<br>taagctggggatcctctagaggttgaggtgattttatgaaaaagaatatcgcatttcttcttgcatctatgttcgtttttctattgctacaaa<br>cgcgtacgctcaggtgcaacttcagcagagcggcgctgaagtcaaaaagcccggatcaagtgtgcgtgtgtcatgcaaagcaagt<br>ggtgggacctttaataataatgcaataaactgggttcggcaggccccgggtcagggactggaatggatgggtggtatcattcctatg<br>tttggaacggcaaaatactctcaaaacttccaaggtcgtgttgcgattactgctgatgagtctacaggtacggcaagcatggaattaa<br>gctctttgcgtagtgaagacacagcggtatattattgtgctcgctccagagatttactgttatttccgcatcatgcattatcgccatgggg<br>tcgtggtaccatggttactgtatcatccgcctccaccaagggcccatcggtcttccccctggcaccctcctccaagagcacctctgg<br>gggcacagcggccctgggctgcctggtcaaggactacttccccgaaccggtgacggtgtcgtggaactcaggcgccctgacca<br>gcggcgtgcacaccttcccggctgtcctacagtcctcaggactctactccctcagcagcgtggtgactgtgccctctagcagcttgg<br>gcacccagacctacatctgcaacgtgaatcacaagcccagcaacaccaaggtggacaagaaagttgagcccaaatcttgtgacaa<br>aactcaccatcaccatcaccattaataa
